# Replication of Type 2 Diabetes Candidate Genes Variations in Three Geographically Unrelated Indian Population Groups

**DOI:** 10.1371/journal.pone.0058881

**Published:** 2013-03-19

**Authors:** Shafat Ali, Rupali Chopra, Siddharth Manvati, Yoginder Pal Singh, Nabodita Kaul, Anita Behura, Ankit Mahajan, Prabodh Sehajpal, Subash Gupta, Manoj K. Dhar, Gagan B. N. Chainy, Amarjit S. Bhanwer, Swarkar Sharma, Rameshwar N. K. Bamezai

**Affiliations:** 1 National Centre of Applied Human Genetics, School of Life Sciences, Jawaharlal Nehru University, New Delhi, India; 2 Shri Mata Vaishno Devi University, Katra, J&K, India; 3 Guru Nanak Dev University, Amritsar, Punjab, India; 4 Department of Biotechnology, Utkal University, Bhubaneshwar, Odisha, India; 5 Jammu University, Jammu, J&K, India; 6 Texas Scottish Rite Hospital, Dallas, Texas, United States of America; 7 School of Biology and Chemistry, Shri Mata Vaishno Devi University, Katra, J&K, India; Yale School of Public Health, United States of America

## Abstract

Type 2 diabetes (T2D) is a syndrome of multiple metabolic disorders and is genetically heterogeneous. India comprises one of the largest global populations with highest number of reported type 2 diabetes cases. However, limited information about T2D associated loci is available for Indian populations. It is, therefore, pertinent to evaluate the previously associated candidates as well as identify novel genetic variations in Indian populations to understand the extent of genetic heterogeneity. We chose to do a cost effective high-throughput mass-array genotyping and studied the candidate gene variations associated with T2D in literature. In this case-control candidate genes association study, 91 SNPs from 55 candidate genes have been analyzed in three geographically independent population groups from India. We report the genetic variants in five candidate genes: *TCF7L2, HHEX, ENPP1, IDE* and *FTO*, are significantly associated (after Bonferroni correction, p<5.5E−04) with T2D susceptibility in combined population. Interestingly, SNP rs7903146 of the *TCF7L2* gene passed the genome wide significance threshold (combined P value = 2.05E−08) in the studied populations. We also observed the association of rs7903146 with blood glucose (fasting and postprandial) levels, supporting the role of *TCF7L2* gene in blood glucose homeostasis. Further, we noted that the moderate risk provided by the independently associated loci in combined population with Odds Ratio (OR)<1.38 increased to OR = 2.44, (95%CI = 1.67–3.59) when the risk providing genotypes of *TCF7L2, HHEX, ENPP1* and *FTO* genes were combined, suggesting the importance of gene-gene interactions evaluation in complex disorders like T2D.

## Introduction

The prevalence of type 2 diabetes (T2D), a complex disorder, is increasing at an alarming rate and becoming a major health problem. The highest incidence of T2D is seen in developing countries where 80% of deaths occur due to diabetes [1]. It has been proposed that the highest number of diabetic patients would be in Asia by the year 2025 [2], [3]. The increased prevalence of type 2 diabetes (T2D) is thought to be due to environmental factors, acting on genetically susceptible individuals [4]. The heritability of T2D is one of the best established among common diseases [5], [6], and consequently, genetic risk factors for T2D have been the subject of intense research [7]. Linkage studies have reported many T2D-linked chromosomal regions and have identified putative, causative genetic variants in *CAPN10, ENPP1, HNF4A, WFS1 and ACDC* [8]–[10]. In parallel, candidate-gene association studies have reported many T2D-associated loci, with coding variants in the nuclear receptor *PPARG* (P12A) [11] and the potassium channel *KCNJ11* (E23K) [12] being among the very few that have been replicated in most of the populations. Multiple genome wide association studies identified the genes including *TCF7L2*, as well as a non-synonymous SNP in the zinc transporter *SLC30A8* and variants in *HHEX, CDKAL1, IGF2BP2* and *CDKN2A/B* [13]–[17]. Study by WTCCC involving a common set of controls for the seven UK wide case cohorts led to the finding of *FTO* to be associated with T2D through its effect on body mass index (BMI) [18]. The Diabetes and Genetics Replication and Meta-Analysis (DIAGRAM) consortium was formed to carry out meta-analyses of three of the previously published studies; WTCCC, DGI and FUSION [13]–[15]. This international collaborative effort identified six new loci *JAZF1, CDC123-CAMK1D, TSPAN8-LGR5, THADA, ADAMTS9* and *NOTCH2* [19]. Few studies from East-Asian ancestry identified additional loci, *KCNQ1, PTPRD, SRR, 13q13.1, UBE2E2* and *CDC4A, CDC4B* associated with T2D [20]–[24]. However, a majority of loci show association in some populations but did not replicate in others, most plausibly a result of genetic heterogeneity in T2D, instead of all being false positive associations. India comprises one of the largest global populations, which had 62.4 million people with type 2 diabetes in year 2011. International Diabetes Federation has predicted it to be 100 million people by year 2030 [25]. It makes it important to replicate and evaluate the previously associated candidates to identify common T2D associated variations/genes, as well as identify novel genetic variations in various Indian population groups to understand the extent of genetic heterogeneity. In the present study, we tried to address it and analyzed 91 SNPs from 55 candidate genes, most of which are previously associated with T2D susceptibility (Table S1) in different world-populations, in three geographically isolated Indian population groups.

## Results and Discussion

Type 2 Diabetes (T2D) is a syndrome of multiple metabolic disorders. It includes abnormally high blood glucose levels (hyperglycemia); involving insulin resistance related signaling pathways and defects in insulin-mediated glucose uptake in muscle; impaired insulin secretion due to dysfunction of pancreatic β cells; disruption of secretary function of adipocytes and an impaired insulin action in liver. The etiology of human T2D involves a strong genetic background [26]. Various approaches including the linkage, candidate gene and the recent genome-wide-studies have been successful to identify more than 40 common genetic variants associated with T2D [27], [28]. These gene variants are related to different metabolic pathways in the disease [29]. However, the total genetic variants roughly account for 10% of the heritability of T2D, suggesting that much remains to be discovered [30]. There is a need to replicate previously associated loci in multiple populations of the world, specifically in Asia including India, where relatively fewer studies have been carried out to identify the common global T2D associated variations/genes; and simultaneously assess the genetic heterogeneity among different population groups for these loci.

The three different population groups of India (Punjab; Jammu and Kashmir; Orissa) recruited in this study were genotyped for 91 SNPs from 55 candidate genes, including those previously associated with T2D susceptibility (Table S1). IBS (Identity by state) analysis (Table S2) showed no significant difference among the cases and controls, in any of the three studied population sets, suggesting those as homogeneous population groups. The detailed description of SNPs, status of Hardy Weinberg equilibrium, allelic frequencies in cases and controls is provided in Table S3. Univariate analysis identified strong association of five genes with the susceptibility to diabetes (Table 1 and Table S4).

**Table 1:** Significant p value (Allelic), odds ratio and risk allele frequency of the SNPs that passed the threshold (combined p<5.0E−04) in three studied populations of India.

SNP	Chr: bp position	Gene	Punjab			Jammu and Kashmir			Orissa			Combined Population	
			Risk Allele Freq			Risk Allele Freq			Risk Allele Freq			Meta-analysis (Fix effect model)	
			Cases/Controls	P value	OR(95% CI)	Cases/Controls	P value	OR(95% CI)	Cases/Controls	P value	OR(95% CI)	P value	OR
**rs7903146_C/T**	**10∶114758349**	**TCF7L2**	0.359/0.303	0.003278	1.28(1.08–1.52)	0.363/0.277	0.0004352	1.48(1.19–1.85)	0.34/0.263	0.0006004	1.44(1.17–1.78)	2.05E–08	1.3833
**rs12255372_G/T**	**10∶114808902**	**TCF7L2**	0.317/0.26	0.001723	1.32(1.11–1.57)	0.31/0.25	0.01045	1.34(1.07–1.69)	0.26/0.206	0.008436	1.35(1.08–1.70)	1.46E–06	1.3376
**rs1887922_T/C**	**10∶94224165**	**IDE**	0.178/0.141	0.01149	1.32(1.06–1.63)	0.2/0.145	0.005809	1.47(1.11–1.93)	0.171/0.14	0.08236	1.26(0.97–1.64)	5.44E–05	1.3423
**rs1111875_A/G**	**10∶94462882**	**HHEX**	0.5/0.45	0.01414	1.22(1.04–1.42)	0.432/0.533	8.94E–05	1.50(1.23–1.84)	0.472/0.441	0.1985	1.13(0.93–1.37)	1.44E−05	1.262
**rs5015480_T/C**	**10∶94465559**	**HHEX**	0.487/0.441	0.02151	1.20(1.02–1.41)	0.452/0.573	5.93E−06	1.63(1.32–2.01)	0.481/0.438	0.07905	1.18(0.98–1.44)	1.83E−06	1.2943
**rs1044498_A/C**	**6∶132172368**	**ENPP1**	0.189/0.144	0.003242	1.37(1.11–1.70)	0.201/0.153	0.01724	1.39(1.05–1.82)	0.188/0.156	0.0864	1.24(0.96–1.61)	4.24E−05	1.341
**rs9939609_T/A**	**16∶53820527**	**FTO**	0.351/0.293	0.001944	1.30(1.10–1.54)	0.369/0.323	0.06208	1.22(0.98–1.52)	0.369/0.306	0.00673	1.32(1.08–1.62)	7.38E−06	1.2906
**rs3751812_G/T**	**16∶53818460**	**FTO**	0.349/0.292	0.002282	1.30(1.09–1.54)	0.372/0.325	0.05986	1.22(0.99–1.52)	0.37/0.313	0.01348	1.29(1.05–1.58)	1.52E−05	1.2786

This study identified the genetic variants in five candidate genes passing the Bonferroni correction (p<5.50E−04) in combined population (Table 1). Interestingly these genes, transcription factors (*TCF7L2, HHEX*), insulin degrading enzyme (*IDE*), fat mass and obesity associated genes (*ENPP1* and *FTO*), showed a consistent association with diabetes susceptibility, involving identical model and risk alleles, as reported earlier in literature. In meta-analysis, Cochrane’s Q test statistic, a test of heterogeneity among the studies, which was not significant for all these SNPs (except rs5015480 of HHEX gene with marginal significance, p = 0.049) also showed consistent association in fixed-effect as well as random effects model. *TCF7L2, HHEX* and *IDE* genes are located at chromosome 10q23-25, the region which has shown strong linkage peak in various genome-wide linkage studies [31], [32]; and was replicated in genome-wide association and candidate gene studies [16],[17]. We wanted to explore whether the association signal is independent of each other or these genes are in linkage disequilibrium (LD). LD analysis of 14 SNPs of *TCF7L2, IDE, HHEX* along with *SIRT1* genes located at chromosome 10q23-25 was performed using Haploview software. Interestingly, the LD analysis showed a very weak or no LD between SNPs from these genes (Figure S1), suggesting an independent risk effect of each locus. These loci remained significant in logistic regression analysis after adjustment with BMI, age and gender as covariates, (Table S5). Along, with the common disease associated variations, shared by all the groups, we observed some variations showing association in population specific manner (p<0.05) and are provided in Table S4. These variations either represent the genetic heterogeneity among the populations or are some false positives, which warrant screening in larger sample sets.

Further, analysis of studied SNPs, using one way Anova and linear correlation, with epidemiological/clinical parameters of diabetes [waist to hip circumference ratio (WHR), BMI, Blood glucose fasting/Postprandial] showed a significant association of SNP, rs7903146 of *TCF7L2* gene with blood glucose (fasting as well as postprandial) in combined population (Table S6), probably indicating that Indian diabetic patients commonly fall in the category of being deficient in Insulin secretion rather than having insulin resistance [33], [34]. TCF7L2, a transcription factor, is reported to be involved in glucose homeostasis, insulin secretion and biosynthesis through GLP1 and wingless type (wnt) signaling pathway is also involved in developmental and growth regulatory mechanism of cells [35]–[37].

The other associated SNPs in this study also belong to important genes that play an important role in various metabolic pathways of diabetes pathobiology. HHEX, a hematopoietically expressed homeobox protein is another transcription factor that is suggested to reduce the β cell secretion capacity and sensitivity of insulin [38]. IDE, a major enzyme (Zn^2+^ - regulated metalloproteinase) expressed ubiquitously including all insulin-responsive tissues responsible for insulin degradation and thereby influencing the extent of the cellular response to insulin [39], [40]. The substrate specificity of IDE coincides with peptides capable of amyloid formation, and may prevent accumulation of amyloidogenic peptides. Disruption of this scavenging function might promote aggregation of the islet amyloid, a characteristic of type 2 diabetes [41]. Interestingly, some other studies have shown the risk allele of rs1887922 in association with increased post loading hyper-insulinemia [42]. ENPP1, an ectonucleotide pyrophosphatase phosphodiesterase, has a role in the insulin resistance by directly inhibiting insulin-induced conformational changes of the insulin receptor, thereby affecting its activation and downstream signaling, which resulted in fasting hyper-insulinemia, a strong predictor for the subsequent development of obesity in children [9], [43]. *FTO* gene variant has been strongly associated with predisposition to diabetes through an effect on BMI [18], [44]. We wanted to see if these biologically related and significantly associated candidate genes show any interactive affect in association to T2D, hence we evaluated interaction in the significantly associated SNPs. The risks provided by the independent loci in our study were moderate (OR<1.38, in combined population, at all loci) as has been shown in other studies [7]. Interaction analysis of genotype combinations of these SNPs with diabetes susceptibility showed an increased effect in associations. We observed that the pair-wise interaction analyses followed by multiple gene interactions (Table S7) shows an increased risk (p = 4.52E−06, OR = 2.44, 95%CI = 1.67–3.59) when the risk providing genotypes of *TCF7L2, HHEX, ENPP1* and *FTO* genes were combined (distributed in 7.24% of patients compared to 3.08% of controls). An increased protection (p = 2.68E−09, OR = 0.28, 95%CI = 0.19–0.43) was also observed for the protection providing genotype combination of *IDE, HHEX, ENPP1* and *FTO* genes (present in 7.63% of controls as compared to 2.12% patients). The observations suggest the importance of identifying not only novel loci in providing disease risk but also understand the role of other mechanisms including the gene-gene and pathway based interaction between multiple functionally important genes, in complex diseases like T2D.

In conclusion, our study suggests *TCF7L2, HHEX, IDE, ENPP1* and *FTO* as commonly associated T2D susceptibility genes in the three Indian populations. Interaction analyses have shown an increased effect in associations suggesting the importance of gene-gene and pathway based interaction between multiple functionally important genes. This study also highlights the importance of multiple population groups based studies in identifying common disease causing genes. Genetic heterogeneity and phenocopies are among the vagaries of complex disorders like T2D, which make understanding of such diseases challenging. It is anticipated that sub-categorization of sample sets by clinical parameters as well as by social groupings like religions castes, etc. and studies of larger data sets will help us better understand the genetic heterogeneity, in complex diseases like T2D especially in Indian populations, our perspective of future studies.

## Materials and Methods

### Ethics Statement

A written informed consent was obtained from all the participants. The data were analysed anonymously, and the study was approved by the ethical committee of Jawaharlal Nehru University.

### Subjects

In the present study, a total of 2900 samples, independent from our previous studies [45], including 1583 well characterized diabetes patients and 1317 controls belonging to three geographically independent population groups of India (649 patients and 600 controls from Punjab, 507 patients and 300 controls from Jammu and Kashmir; 427 patients and 417 controls from Orissa), were included. Diagnosis of T2D was made according to the criteria of World Health Organization (Expert Committee 2003). The patients with a history of ketoacidosis/requiring continuous insulin treatment since diagnosis/having exocrine pancreatic disease/or with exceptionally early age of onset (<30 years), were excluded. Patients with severe liver or renal dysfunction were also excluded. Non-diabetic individuals with no known positive family history of diabetes were included in the study. The studied individuals were confirmed of being unrelated for three generations. Anthropometric measurements and other features are summarized in Table S8.

### Assessment of the Clinical Parameters

The patient was diagnosed with hypertension when the systolic blood pressure (SBP) was 140 mmHg and the diastolic blood pressure (DBP), 90 mmHg. Overweight and Obese together were clinically characterized by body mass index (BMI) of >24.9 (BMI is defined by ratio of weight in kilograms to square of height in meters) and the increased abdominal fat was measured by waist to hip circumference ratio (WHR) of 0.94.

### SNP Selection and Genotyping

SNPs in this study (Table S1) were included from those genes which have been implicated with T2D or diabetes related traits through genome-wide association studies, mostly in European populations, and further replicated in other populations using candidate gene approach [13], [16], [17], [46]–[57]. In addition, other gene SNPs that have been studied with T2D susceptibility but not replicated or studied in multiple populations were also included. Genotyping of SNPs was performed using High-throughput genotyping MassArray platform (SEQUENOM) as described earlier [58]. SNP genotyping success rate was >95%. For quality control of SNP genotyping, each 96 well plate contained three or more duplicate samples and a negative control. The concordance rate for genotyping was >99.5%.

### Statistical Analyses

Statistical analyses were mainly performed using PLINK v. 1.07 (http://www. pngu.mgh.harvard.edu/purcell/plink/). Each SNP was tested for Hardy Weinberg Equilibrium. Pairwise IBS (Identity by state) distances between all individuals have also been calculated with respect to binary phenotype (non-significant SNPs of this study only), to know if there are hints of group differences. IBS analysis is most robust for genome-wide data; however, this analysis provides a preliminary evidence of no population stratification. Significant association of SNPs was tested by 3×2 Chi square test for overall genotype frequency distributions between diabetes patients and controls. Association of SNP with type 2 diabetes was further confirmed by conditional logistic regression analysis with forward conditional method adjusted for possible confounding factors: age, gender and BMI. Odds ratios (ORs) were calculated with respect to risk allele. Meta-analysis was performed by combining summary estimates both under random effect and fixed effect models using PLINK v. 1.07, which also provides Cochrane’s Q test statistic, a test of heterogeneity among the studies, P value <5.5×10^−4^ (0.05/91)) was considered significant after Bonferroni correction. We also explored genotypic interactions of significantly associated SNPs using logistic regression with forward conditional method. These analyses were performed using statistical software SPSS v20.0 (SPSS, Chicago III, IL, USA.

Association of SNPs with quantitative traits was determined using one way ANOVA adjusted for age, sex, population and BMI as appropriate, in control, patients and total population. P value <2.77×10^−4^ (0.05/180) was considered significant after Bonferroni correction (10 SNPs×6 parameters in 3 population groups). Epidemiological parameters [Age, Waist/Hip ratio, BMI, Gender, Systolic Blood pressure, Diastolic Blood pressure] were compared between patients and controls using linear regression analysis. Statistical power of the study was estimated using QUANTO version 1.2 (http://hydra.usc.edu/gxe/). Sample size included in this study had 70–97% power to detect the association with OR of 1.3–1.5 assuming minor allele frequency of 0.20.

## Supporting Information

Figure S1 **Linkage disequilibrium (LD) analysis (r^2^ value) of 14 SNPs of** ***TCF7L2, IDE, HHEX*** **and** ***SIRT1*** **genes located at chromosome 10q23-25.**
(TIF)

Table S1 **Details of the SNPs selected for present study.**
(XLS)

Table S2 **Permutation test for: between group (case-control) Identity by State (IBS) difference with respect to binary phenotype for independent analysis of three populations.**
(DOC)

Table S3 **Characteristic of 91 SNPs from 55 genes studied in three different populations (Punjab; Jammu and Kashmir; and Orissa) of India.**
(XLS)

Table S4 **Allele frequencies, P values, odds ratios of the studied SNPs in three studied populations of India.**
(XLSX)

Table S5 **Logistic regression analysis of most significantly associated SNPs of each associated gene, adjusted with age, gender and BMI in combined population.**
(DOC)

Table S6 **One Way ANOVA of blood glucose (fasting and post prandial) with** ***TCF7L2*** **gene SNPs.**
(DOC)

Table S7 **Genotype Interaction analysis of significantly associated SNPs.**
(DOC)

Table S8 **Distribution of epidemiological parameters between diabetes patients and Controls populations for studied three populations of India.**
(DOC)
