# Supplementary figures and images for: Replication of Type 2 Diabetes Candidate Genes Variations in Three Geographically Unrelated Indian Population Groups
Source: PLoS One. 2013 Mar 19;8(3):e58881. doi: 10.1371/journal.pone.0058881 (PMC3602599; doi:10.1371/journal.pone.0058881)

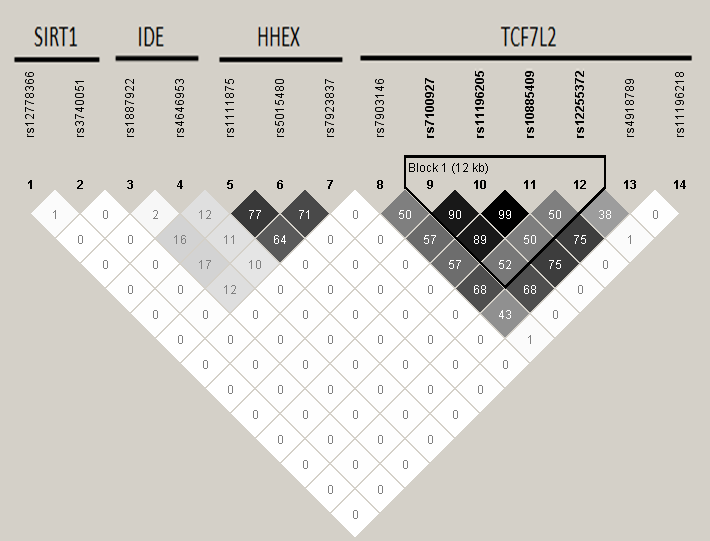

Supplement: Figure S1 — Linkage disequilibrium (LD) analysis (r2 value) of 14 SNPs of TCF7L2, IDE, HHEX and SIRT1 genes located at chromosome 10q23-25. (TIF) [file pone.0058881.s001.tif]
