# Supplementary material for: Replication of Type 2 Diabetes Candidate Genes Variations in Three Geographically Unrelated Indian Population Groups
Source: PLoS One. 2013 Mar 19;8(3):e58881. doi: 10.1371/journal.pone.0058881 (PMC3602599; doi:10.1371/journal.pone.0058881)
Supplement: Table S2 — Permutation test for: between group (case-control) Identity by State (IBS) difference with respect to binary phenotype for independent analysis of three populations. (DOC) [file pone.0058881.s003.doc]

**Supplementary table S2**: Permutation test for: between group (case-control) Identity by State (IBS) difference with respect to binary phenotype for independent analysis of three populations.

|  | **Orissa** | **Jammu and Kashmir** | **Punjab** |
| --- | --- | --- | --- |
| Between-group IBS (mean, SD) | 0.737634, 0.0410635 | 0.731804, 0.041025 | 0.735303, 0.0404921 |
| In-group (2) IBS (mean, SD) | 0.736866, 0.041203 | 0.730433, 0.0416237 | 0.736572, 0.0398543 |
| In-group (1) IBS (mean, SD) | 0.738158, 0.0415173 | 0.73311, 0.0409613 | 0.734158, 0.0413651 |
| T1: Case/control less similar (p value) | 0.895891 | 0.870071 | 0.0738893 |
| T2: Case/control more similar (p value) | 0.104119 | 0.129939 | 0.926121 |
