# Supplementary material for: Replication of Type 2 Diabetes Candidate Genes Variations in Three Geographically Unrelated Indian Population Groups
Source: PLoS One. 2013 Mar 19;8(3):e58881. doi: 10.1371/journal.pone.0058881 (PMC3602599; doi:10.1371/journal.pone.0058881)
Supplement: Table S5 — Logistic regression analysis of most significantly associated SNPs of each associated gene, adjusted with age, gender and BMI in combined population. (DOC) [file pone.0058881.s006.doc]

**Supplementary Table S5:** Logistic regression analysis of most significantly associated SNPs of each associated gene, adjusted with age, gender and BMI in combined population.

| **Variables** | **B** | **S.E.** | **Wald** | **df** | **Sig.** | **OR(95%CI)** |
| --- | --- | --- | --- | --- | --- | --- |
| **rs7903146** | .373 | .080 | 21.480 | 1 | 3.58E-06 | 1.45(1.23-1.69) |
| **rs1887922** | .228 | .094 | 5.849 | 1 | .0155825 | 1.25(1.04-1.51) |
| **rs5015480** | .453 | .095 | 22.595 | 1 | 2.00E-06 | 1.57(1.30-1.89) |
| **rs1044498** | .412 | .087 | 22.616 | 1 | 1.98E-06 | 1.51(1.27-1.78) |
| **rs9939609** | .399 | .081 | 24.391 | 1 | 7.86E-07 | 1.49(1.27-1.74) |
