# Supplementary material for: Replication of Type 2 Diabetes Candidate Genes Variations in Three Geographically Unrelated Indian Population Groups
Source: PLoS One. 2013 Mar 19;8(3):e58881. doi: 10.1371/journal.pone.0058881 (PMC3602599; doi:10.1371/journal.pone.0058881)
Supplement: Table S6 — One Way ANOVA of blood glucose (fasting and post prandial) with TCF7L2 gene SNPs. (DOC) [file pone.0058881.s007.doc]

**Supplementary Table S6:** One Way ANOVA of blood glucose (fasting and post prandial) with *TCF7L2* gene SNPs.

|  | **Genotype** | **Combined Population** | | | **Combined Patients** | | | **Combined Controls** | | |
| --- | --- | --- | --- | --- | --- | --- | --- | --- | --- | --- |
|  |  | **Mean/±SE** | **p value** | | **Mean/±SE** | **p value** | | **Mean/±SE** | **p value** | |
|  | |  | **Unadjusted** | **Adjusted*** |  | **Unadjusted** | **Adjusted*** |  | **Unadjusted** | **Adjusted*** |
| **rs7903146** |  |  |  |  |  |  |  |  |  |  |
| Blood glucose  (fast) mg/dl | CC | 131.07/±2.07 | 5.50E-06 | 0.00022 | 151.90/±2.42 | 0.002 | 0.028 | 84.10/±0.81 | 0.031 | 0.56 |
|  | CT | 147.59/±2.7 |  |  | 165.60/±2.99 |  |  | 82.49/±0.84 |  |  |
|  | TT | 140.94/±4.18 |  |  | 157.23/±4.61 |  |  | 87.80/±2.14 |  |  |
| Blood glucose  (PP) mg/dl | CC | 182.18/±2.97 | 1.42E-05 | 2.9E-06 | 213.87/±3.25 | 0.005 | 0.003 | 107.87/±1.9 | 0.95 | 0.888 |
|  | CT | 197.84/±3.52 |  |  | 226.85/±3.86 |  |  | 108.28/±1.76 |  |  |
|  | TT | 211.75/±6.37 |  |  | 234.76/±6.48 |  |  | 106.93/±2.74 |  |  |
| **rs12255372** | |  |  |  |  |  |  |  |  |  |
| Blood glucose  (fast) mg/dl | GG | 135/±2.06 | 0.021 | 0.016 | 155.72/±2.38 | 0.240 | 0.166 | 84.11/±0.79 | 0.095 | 0.817 |
|  | GT | 144.01/±2.68 |  |  | 161.89/±2.97 |  |  | 82.27/±0.94 |  |  |
|  | TT | 142.25/±5.24 |  |  | 161.07/±5.9 |  |  | 86.93/±2.29 |  |  |
| Blood glucose  (PP) mg/dl | GG | 185.37/±2.77 | 2.97E-04 | 0.000093 | 216.74/±3.01 | 0.006 | 0.005 | 107.84/±1.75 | 0.80 | 0.894 |
|  | GT | 196.7/±3.68 |  |  | 224.58/±4.05 |  |  | 108.39/±1.83 |  |  |
|  | TT | 214.28/±7.69 |  |  | 242.53/±7.73 |  |  | 105.10/±2.41 |  |  |

* p value adjusted with age, gender, BMI and population.
