# Supplementary material for: Replication of Type 2 Diabetes Candidate Genes Variations in Three Geographically Unrelated Indian Population Groups
Source: PLoS One. 2013 Mar 19;8(3):e58881. doi: 10.1371/journal.pone.0058881 (PMC3602599; doi:10.1371/journal.pone.0058881)
Supplement: Table S7 — Genotype Interaction analysis of significantly associated SNPs. (DOC) [file pone.0058881.s008.doc]

**Supplementary Table S7:** Genotype Interaction analysis of significantly associated SNPs.

| **Interactions** | **TCF7L2** | | **IDE** | **HHEX** | | | **ENPP1** | **FTO** | | **B** | **Sig p value** | | **OR(95%CI)** |
| --- | --- | --- | --- | --- | --- | --- | --- | --- | --- | --- | --- | --- | --- |
|  | **rs7903146** | **rs12255372** | **rs1887922** | **rs1111875** | **rs5015480** | **rs7923837** | **rs1044498** | **rs9939609** | **rs3751812** |  |  |  |  |
| **1** | TT+CT | TT+GT |  | GG+AG | CC+TC | GG+AG | AA | AA+AT | TT+GT | 0.28 | 0.02403 | 1.32(1.03-1.69) | |
| **2** | TT+CT | TT+GT |  | GG+AG | CC+TC | GG+AG | CC+CA | AA+AT | TT+GT | 0.90 | 4.52E-06 | 2.44(1.66-3.58) | |
| **3** | CC | GG |  | AA | TT | AA | AA | TT | GG | -2.5 | 7.08E-07 | 0.07(0.02-0.2) | |
| **Constant** |  |  |  |  |  |  |  |  |  | 0.18 | 0.00004 |  | |
|  |  |  |  |  |  |  |  |  |  |  |  |  | |
|  |  |  |  |  |  |  |  |  |  |  |  |  | |
| **4** |  |  | CC+CT | GG+GA | CC+TC | GG+GA | CC+CA | AA+AT | GG | 0.29 | 0.02381 | 1.33(1.03-1.72) | |
| **5** |  |  | TT | GG+GA | CC+TC | GG+GA | CC+CA | AA+AT | TT+GT | 0.45 | 0.00471 | 1.57(1.14-2.14) | |
| **6** |  |  | CC+CT | GG+GA | CC+TC | GG+GA | CC+CA | AA+AT | TT+GT | 0.68 | 0.00067 | 1.98(1.33-2.93) | |
| **7** |  |  | TT | AA | TT | AA | AA | TT | GG | -1.2 | 2.68E-09 | 0.28(0.18-0.43) | |
| **8** |  |  | TT | AA | TT | GG+GA | AA | TT | GG | -1.1 | 0.01452 | 0.3(0.12-0.79) | |
| **Constant** |  |  |  |  |  |  |  |  |  | 0.19 | 0.00002 |  | |
