# Supplementary material for: Replication of Type 2 Diabetes Candidate Genes Variations in Three Geographically Unrelated Indian Population Groups
Source: PLoS One. 2013 Mar 19;8(3):e58881. doi: 10.1371/journal.pone.0058881 (PMC3602599; doi:10.1371/journal.pone.0058881)
Supplement: Table S8 — Distribution of epidemiological parameters between diabetes patients and Controls populations for studied three populations of India. (DOC) [file pone.0058881.s009.doc]

**Supplementary Table S8:** Distribution of epidemiological parameters between diabetes patients and Controls populations for studied three populations of India.

| **Parameter** | | **North India[Punjab]** | | | | | **Jammu and Kashmir** | | | | **Orissa** | | | | **Total** | | | |
| --- | --- | --- | --- | --- | --- | --- | --- | --- | --- | --- | --- | --- | --- | --- | --- | --- | --- | --- |
|  |  | **N** | **Mean** | | **S.D./S.E. or (%)** | **Sig. p value** | **N** | **Mean** | **S.D./S.E.**  **or (%)** | **Sig. p value** | **N** | **Mean** | **S.D./S.E.**  **or (%)** | **Sig.p value** | **N** | **Mean** | **S.D./S.E.**  **or (%)** | **Sig.p value** |
| **AGE (Yrs)** | |  |  | |  |  |  |  |  |  |  |  |  |  |  |  |  |  |
| Controls | | 591 | 52.98 | | 13.10/0.539 | 0.034 | 296 | 44.73 | 10.68/0.62 | 1.6E-12 | 417 | 54.12 | 10.713/0.524 | 0.064 | 1304 | 51.47 | 12.40/0.343 | 1.7E-05 |
| Patients | | 642 | 54.42 | | 10.65/0.42 |  | 501 | 50.05 | 9.75/0.435 |  | 427 | 55.37 | 8.81/0.426 |  | 1570 | 53.28 | 10.13/0.255 |  |
| **Gender** | |  |  | |  |  |  |  |  |  |  |  |  |  |  |  |  |  |
| Controls | Male | 246 | | 41.1% | | | 140 |  | 46.7% |  | 248 |  | 59.5% |  | 681 |  | 51.7% |  |
|  | Female | 352 | | 58.9% | | | 160 |  | 53.3% |  | 169 |  | 40.5% |  | 634 |  | 48.1% |  |
| Patients | Male | 303 | | 47.0% | | | 281 |  | 55.5% |  | 269 |  | 63.0% |  | 725 |  | 45.8% |  |
|  | Female | 342 | | 53.0% | | | 225 |  | 44.5% |  | 158 |  | 37.0% |  | 853 |  | 53.9% |  |
| **Hypertension (>140/90 mmHg)** | |  |  | |  |  |  |  |  |  |  |  |  |  |  |  |  |  |
| Controls | | 103 |  | | 17.3% |  | 32 |  | 10.8% |  | 35 |  | 8.4% |  | 170 |  | 13.0% |  |
| Patients | | 90 |  | | 14.0% |  | 118 |  | 23.3% |  | 64 |  | 15.2% |  | 272 |  | 17.3% |  |
| **BMI ~~CODE~~** | |  |  | |  |  |  |  |  |  |  |  |  |  |  |  |  |  |
| Controls | | 591 | 24.63 | | 4.499/0.185 | 7.6E-14 | 297 | 25.74 | 4.27/0.248 | 0.021 | 417 | 24.43 | 4.086/0.2 | 0.0068 | 1305 | 24.82 | 4.347/0.12 | 1.3E-09 |
| Patients | | 641 | 26.72 | | 5.135/0.202 |  | 507 | 26.54 | 5.01/0.222 |  | 427 | 23.77 | 2.8805/0.139 |  | 1575 | 25.86 | 4.76/0.119 |  |
| **WHR** | |  |  | |  |  |  |  |  |  |  |  |  |  |  |  |  |  |
| Controls | | 598 | 0.916 | | 0.076/0.003 | 8.5E-12 | 39 | 1.010 | 0.188/0.03 | 0.138 | 416 | 0.95 | 0.0857/0.004 | 0.00036 | 1053 | 0.93 | 0.089/0.002 | 3.2E-03 |
| Patients | | 641 | 0.947 | | 0.084/0.003 |  | 124 | 0.980 | 0.0702/0.006 |  | 425 | 0.93 | 0.09/0.004 |  | 1190 | 0.94 | 0.086/0.002 |  |
| **Blood Glucose (fasting)mg/dl** | |  |  | |  |  |  |  |  |  |  |  |  |  |  |  |  |  |
| Controls | | 103 | 89.71 | | 18.75/1.847 | 3.6E-28 | 300 | 81.60 | 7.471/0.431 | 2.77E-91 | - | | |  | 403 | 83.67 | 11.97/0.596 | 1.1E-114 |
| Patients | | 250 | 175.83 | | 71.55/4.525 |  | 501 | 159.8 | 58.14/2.597 |  | 401 | 147.23 | 52.827/2.638 |  | 1152 | 158.9 | 60.46/1.781 |  |
| **Blood Glucose (PP) mg/dl** | |  |  | |  |  |  |  |  |  |  |  |  |  |  |  |  |  |
| Controls | | 181 | 108.98 | | 31.33/2.328 | 1.1E-50 | 250 | 106.92 | 19.13/1.21 | 8.9E-104 | - | | |  | 431 | 107.78 | 24.98/1.203 | 1.3E-146 |
| Patients | | 394 | 235.23 | | 100.2/5.048 |  | 423 | 230.08 | 73.34/3.566 |  | 401 | 202.12 | 64.989/3.245 |  | 1218 | 222.54 | 81.88/2.346 |  |
| **SBP(mmHg)** | |  |  | |  |  |  |  |  |  |  |  |  |  |  |  |  |  |
| Controls | | 598 | 129.4 | | 18.40/0.752 | 0.089 | 296 | 129.65 | 14.32/0.832 | 4.8E-10 | 417 | 123.73 | 14.091/0.69 | 0.017 | 1311 | 127.65 | 16.45/0.454 | 3.8E-05 |
| Patients | | 645 | 127.77 | | 15.21/0.599 |  | 454 | 137.57 | 18.25/0.856 |  | 427 | 126.45 | 18.581/0.899 |  | 1526 | 130.32 | 17.76/0.454 |  |
| **DBP(mmHg)** | |  |  | |  |  |  |  |  |  |  |  |  |  |  |  |  |  |
| Controls | | 598 | 85.36 | | 10.74/0.439 | 0.010 | 296 | 81.40 | 4.970/0.288 | 0.0060 | 417 | 81.38 | 4.744/0.232 | 0.242 | 1311 | 83.20 | 8.32/0.229 | 0.0169 |
| Patients | | 645 | 87.06 | | 12.09/0.476 |  | 454 | 82.66 | 6.798/0.319 |  | 427 | 80.89 | 6.9685/0.337 |  | 1526 | 84.03 | 9.81/0.251 |  |

N represents the number of individuals. WHR ratio: waist to hip ratio; S.D.: standard deviation; S.E.: standard error; PP: Postprandial;
